# Ehlers-Danlos syndrome versus cleidocranial dysplasia

**DOI:** 10.1186/1824-7288-40-49

**Published:** 2014-05-24

**Authors:** Maria Francesca Bedeschi, Francesca Bonarrigo, Francesca Manzoni, Donatella Milani, Maria Rosaria Piemontese, Sophie Guez, Susanna Esposito

**Affiliations:** 1 Medical Genetic Unit, Fondazione IRCCS Ca' Granda Ospedale Maggiore Policlinico, Milan, Italy; 2 Pediatric Highly Intensive Care Unit, Department of Pathophysiology and Transplantation, Università degli Studi di Milano, Fondazione IRCCS Ca’ Granda Ospedale Maggiore Policlinico, Via Commenda 9, Milan 20122, Italy; 3 Medical Genetics Unit, IRCCS Casa Sollievo della Sofferenza, San Giovanni Rotondo, Italy

## 

Dear Sir,

The early identification of hereditary syndromes is essential for planning medical and surgical interventions for reducing the risk of complications [1]. Unfortunately, clinical phenotypes of hereditary syndromes in the first years of life and in mild cases are often poorly characterized. Some disease symptoms are also common to several different genetic conditions. Cleidocranial dysplasia (CCD, OMIM #119600) is a genetic condition that predominantly affects the skeletal system. Typical CCD features include persistently open skull sutures, clavicular hypoplasia/aplasia, and dental anomalies [2,3]. CCD is caused by a heterozygous loss-of-function mutation in the *RUNX2* gene [2,3]. However, the abnormal shoulder and arm mobility commonly observed in CCD is also typical of other syndromes, particularly hypermobile Ehlers-Danlos syndrome (EDS-HT). EDS-HT is marked by joint laxity with minimal skin changes and no skin fragility [4] but does not have additional specific clinical features and cannot be diagnosed through laboratory tests.

The child characterized in this report was initially misdiagnosed with EDS-HT when the correct diagnosis was CCD. CCD was confirmed by genetic findings but not until several years later. The male proband was the second child born to healthy, non-consanguineous Caucasian parents. The family history was unremarkable and did not indicate a history of mental retardation, genetic diseases, or birth defects. The child was born at 35 weeks of gestational age as the result of a premature membrane rupture after an uneventful pregnancy. At birth, he weighed 2,700 gr and had a body length of 45.5 cm, occipital frontal diameter of 30 cm, and an APGAR score of 9/10.

The patient’s medical history after reaching school age was unremarkable, and he had normal body and psychomotor growth. However, delayed anterior fontanel closure and prolonged deciduous dentition retention were reported. At nine years of age, the patient experienced constant bilateral shoulder dislocation associated with bilateral flat foot and the delayed eruption of permanent teeth. The patient was evaluated by a pediatrician with experience in the clinical genetics field and was diagnosed with EDS-HT.

At 13 years of age, the patient entered the pediatric outpatient clinic of our hospital for shoulder dislocation and pain. He appeared to be in good health with normal physical and neurological development. However, he had minor facial anomalies, such as a broad and flat forehead, hypertelorism, mid-face hypoplasia, and small, spaced teeth (Figure 1). The patient also had slightly smooth thigh and trunk skin, slightly elastic and pasty abdominal skin, normotrophic scars, bilateral shoulder dislocations, mild hyperlaxity of the hands, feet articulations, and bilateral flat foot. In addition, chest X-rays showed the presence of hypoplastic clavicles (Figure 2). These symptoms, together with the facial anomalies, suggested that the patient had CCD, not EDS-HT. We performed additional DNA testing to confirm this diagnosis, but no *RUNX2* point mutations, which are predicted in a third of all CCD cases, were identified [2,3]. However, we identified a *de novo* heterozygous deletion of the *RUNX2* exon 2 using a multiplex ligation-dependent probe amplification (MLPA) kit (P080, MRC- Holland, Amsterdam, The Netherlands), confirming the CCD diagnosis.

**Figure 1:**
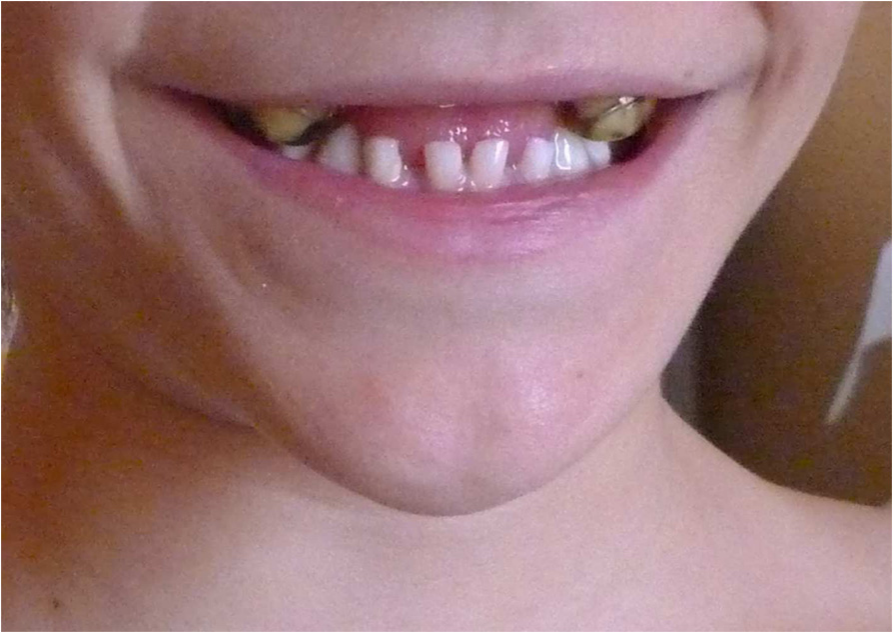
Small and widely spaced teeth.

**Figure 2:**
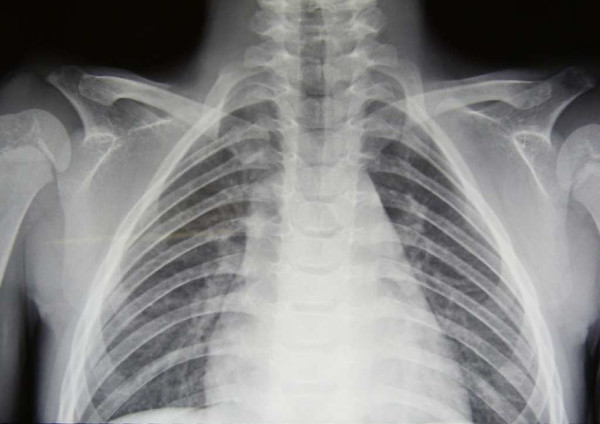
Chest X-ray showing bilateral clavicular hypoplasia.

EDS is characterized by skin, joint, ligament, blood vessel, and internal organ changes [4]. Most cases of severe and continuous joint instability are the result of collagen abnormalities [5]. Thus, continuous shoulder dislocations alone or in the presence of other types of joint instability are considered an essential marker of EDS [6]. This may explain why the child was initially diagnosed with EDS and why no other evaluations were considered necessary.

Standardized and updated clinical diagnostic criteria for EDS-HT and other overdiagnosed asymptomatic joint hypermobility are urgently needed. Joint instability can be a symptom of other conditions, particularly several hereditary connective tissue disorders [4]. In CCD, orthopaedic problems can be caused not only by skeletal features (particularly clavicular hypoplasia) but also by periarticular tissue laxity and altered relations between primary and/or secondary joints [7]. A radiograph of the child’s shoulder when he was initially diagnosed may have shown the hypoplastic clavicles that are strongly characteristic of CCD. The patient’s delayed anterior fontanel closure and delayed permanent teeth eruption are also characteristic of CCD [7] but not EDS [8,9]. Thus, a better evaluation of the patient’s clinical history could have contributed to an earlier CCD diagnosis.

A definitive CCD diagnosis is only possible using genetic analyses. In this case, DNA sequencing did not identify a causative *RUNX2* mutation. However, MLPA was able to detect a deletion in *RUNX* that not only confirmed the correct diagnosis but also provided an accurate estimate of future reproductive risks [10]. A delay in CCD identification can be deleterious since both the parents and patient were informed on an incorrect expected natural disease course. In addition, several potential clinical complications can arise, including: upper airway obstruction with increased risk for sinus and ear infections, dental abnormalities, and relevant orthopaedic problems. Decreases in hearing and osteoporosis can also occur during adolescence.

This case showed how diagnostic approaches to hereditary syndromes must include the patient’s clinical history, signs and symptoms, as well as genetic analyses. A cursory examination can led to misdiagnoses that can impact the clinical course of the actual disease and also the patient’s quality of life.

## Consent

Written informed consent was obtained from the patient’s parents for the publication of this report and any accompanying images. A copy of the written informed consent is available for this journal’s Editor-in-Chief to review.

## Abbreviations

CCD: Cleidocranial dysplasia; EDS: Ehlers-Danlos syndrome; MLPA: Multiplex ligation-dependent probe amplification.

## Competing interests

The authors declare no competing interests.

## Authors’ contributions

MFB and SG clinically diagnosed the patient and were involved in drafting the manuscript, revising it for intellectual content, and approving the final version for publication. FB and FM made substantial contributions to data acquisition. DM helped draft and revise the manuscript. MRP performed the genetic analyses. SE revised the manuscript for intellectual content and approved the final version for publication. All authors have read and approved the final manuscript.
